# Combined Use of Voltage Mapping and Speckle-tracking Analysis for the Characterization of Arrhythmogenic Right Ventricular Cardiomyopathy: A Case Report

**DOI:** 10.19102/icrm.2022.130501

**Published:** 2022-05-15

**Authors:** Amato Santoro, Nicolò Sisti, Claudia Baiocchi, Giulia Elena Mandoli, Antonio Biancofiore, Simone Pistoresi, Valerio Zacà, Salvatore Francesco Carbone, Marta Focardi, Flavio D’Ascenzi, Matteo Cameli

**Affiliations:** ^1^Department of Medical Biotechnologies, Division of Cardiology, University of Siena, Siena, Italy; ^2^Department of Diagnostic Imaging, Azienda Ospedaliera Universitaria Senese, Siena, Italy

**Keywords:** AVRC, Cartosound^®^, intracardiac echocardiography, speckle tracking echocardiography, voltage mapping

## Abstract

A 38-year-old man was admitted to our hospital after ventricular tachycardia. Endocardial bipolar and unipolar voltage mapping were performed and findings were integrated with data from intracardiac echocardiography (ICE) right ventricular (RV) speckle-tracking analysis. A reduction in the strain analysis was stored in correspondence of the fragmented electrogram area. The definitive diagnosis was arrhythmogenic RV cardiomyopathy (ARVC). The integration of ICE-derived RV strain and voltage mapping could represent a successful strategy to improve the results of ablation in ARVC.

## Case presentation

**Figure 1:**
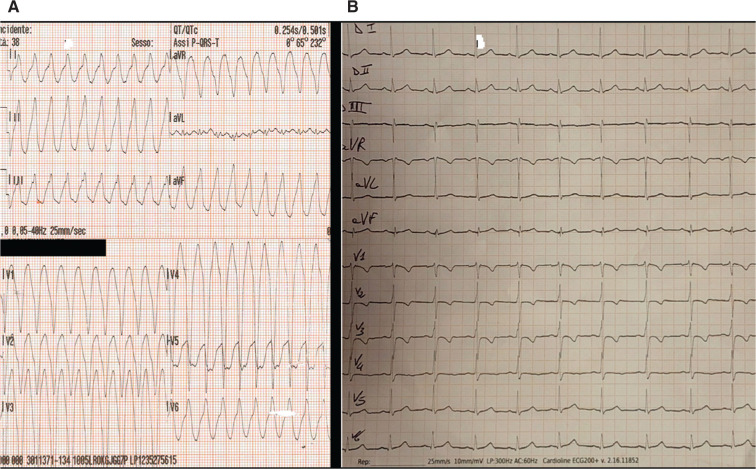
**A:** Electrocardiogram recorded in the emergency setting showing ventricular tachycardia with left bundle branch block and an inferior axis morphology. **B:** Electrocardiogram recorded a few hours later demonstrating an epsilon wave in V1 and inversion of T-waves in V1–V3.

A 38-year-old man was admitted to our hospital after a sustained ventricular tachycardia (VT) with a left bundle branch block (LBBB) morphology with an inferior axis, QRS transition in V5. The cycle length of the VT was 250 ms **(Figure 1A)**. Three hundred milligrams of amiodarone was then administered intravenously, resulting in the restoration of sinus rhythm. Twelve-lead resting electrocardiography (ECG) was performed, which revealed a suspected notch as an epsilon wave at V1 **(Figure 1B)**, with negative T-waves observed in leads V1–V3.

Transthoracic echocardiography (TTE) revealed a right ventricle (RV) with bulging of the free-wall mid-segment from the 4-chamber apical view. Speckle-tracking echocardiography (STE) analysis performed on TTE clips revealed a reduction in RV longitudinal strain (LS) in all 3 myocardial layers in the sub-tricuspid region.

**Figure 2:**
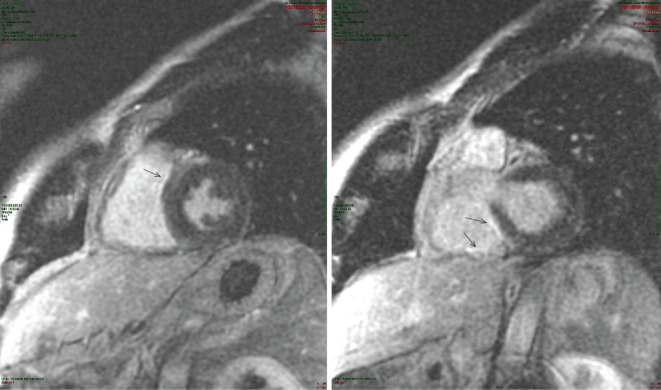
RM scans with late gadolinium enhancement sequences. Arrows indicate late gadolinium enhancements at the level of anterior middle-basal septum (left) and the inferior biventricular junction (right).

During the observation, the patient underwent cardiac magnetic resonance imaging, which showed an RV with slightly reduced ejection fraction (EF) of 45%, dyskinesia of the mid-segment of the free wall, and a non-ischemic subepicardial pattern of late-gadolinium enhancement at the mid-basal anterior septum and inferior biventricular junction **(Figure 2)**.

In-hospital ECG monitoring documented sporadic ventricular premature beats (VPBs), couples, and triplets with 2 different morphologies, the first of which had an LBBB pattern with an inferior axis, comparable to the morphology of the VT previously observed, and the second of which had an LBBB pattern with a superior axis.

According to the 2010 Task Force Criteria,^1,2^ ARVC was diagnosed by the combination of an epsilon wave, negative T-wave from V1–V3 and non-sustained VT with LBBB and a superior axis (major criteria), mildly increased RV dimension, and reduced EF using cardiac magnetic resonance imaging (minor criteria). Genetic testing confirmed the presence of a known pathogenic mutation in the *DSP* gene, a gene in which truncating alleles have been reported when ARVC is present.^3^

## Electrophysiologic study and voltage mapping

**Figure 3:**
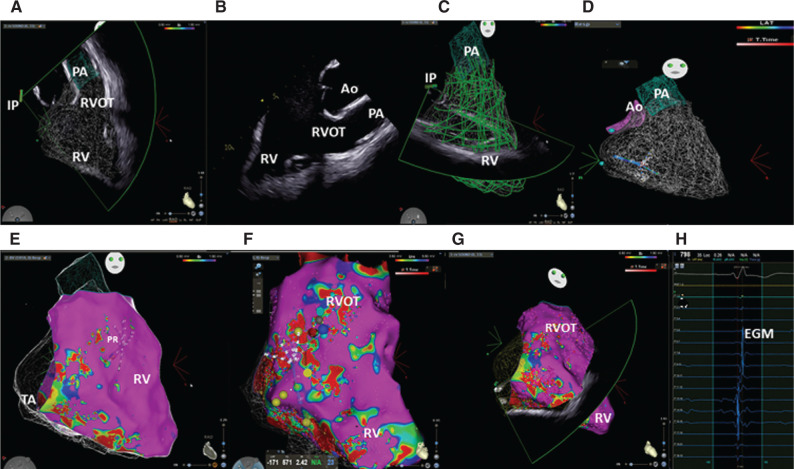
**A:** In the 3-dimensional intracardiac echocardiography (ICE) map, the probe is positioned in the right atrium; this is the ICE home view. **B:** Two-dimensional (2D) ICE visualization of the inner and outer tracts of the right ventricle (RV); this is the 2D home view visualization. **C:** 2D ICE visualization of the RV in the transversal view. The green segments are the beams acquired to obtain a 3D echocardiographic RV map. **D:** 3D ICE map of the RV. A multipolar catheter is in the inner tract of the RV. **E:** A merged ICE and bipolar voltage map is represented. Low-voltage areas were stored at the sub-tricuspidalic annulus and at the inferior apex of the RV. **F:** A merged ICE and voltage unipolar map is represented. It shows the low-voltage areas in the anteroseptal outer tract. **G:** A merged ICE and voltage bipolar map; real-time ICE probe beam of the RV during voltage mapping. **H:** Low potential stored. *Abbreviations:* Ao, aorta; IP, ice probe; PA, pulmonary artery; RA, right atrium; RVOT, right ventricular outer tract; TA, tricuspid annulus.

The patient underwent an invasive electrophysiologic (EP) study by a dedicated mapping software and intracardiac echocardiography (ICE), obtaining a 3-dimensional (3D) map. Two morphologies of VPBs were identified at baseline during sinus rhythm, originating from the RV inferior wall and the RV outflow tract (RVOT), respectively. In the ICE 3D-RV reconstruction, the multipolar catheter performed a voltage map reconstruction without fluoroscopy **(Figures 3A–3D)**, and a 3D fast anatomical map was then created and merged with the 3D echo map. Bipolar voltage definitions of abnormal and normal myocardium were based on previously validated data in the RV **(Figure 3E)**. Detailed maps of the endocardial RV surface during sinus rhythm were obtained with a dedicated electroanatomic mapping (EAM) system and a 4-mm standard tip catheter or 3.5-mm open irrigated-tip catheter and multipolar catheter using bipolar (bandpass-filtered at 10–400 Hz) and unipolar (bandpass-filtered at 1–240 Hz) voltage mapping. The endocardial regions with a bipolar electrogram amplitude > 1.5 mV were defined as “normal,” “scar” was defined as an area with an amplitude < 0.5 mV, and “abnormal” myocardium was defined as a region with a bipolar electrogram amplitude of 0.5–1.5 mV or unipolar amplitude between 3.5–5.5 mV **(Figure 3F)**. The voltage map was obtained using a total of 6,000 map points, and unipolar and bipolar voltage mapping of the RV showed low-voltage areas and corresponding fragmented potentials from the tricuspid annulus to the inferior apex **(Figures 3G and 3H)**. In the RVOT, the bipolar voltage mapping was normal, while the unipolar mapping showed low-voltage areas in the anteroseptal outflow tract. An offline map was also used to perform STE on intracardiac RV and standard echocardiography (TTE).

Pace-mapping found a 97% match for the morphology from the inferior wall and a 95% match for the morphology from the RVOT. Isoprenaline infusion was then started, inducting sinus tachycardia and aberrant atrioventricular conduction with an RBBB and superior axis morphology. After a programmed ventricular stimulation (drive of 600, 500, or 400 with 1, 2, or 3 extra-stimuli from the RV apex and RVOT, respectively, until the RV refractory period), non-sustained VT was induced with both an LBBB pattern and inferior axis and an LBBB pattern and superior axis. With TTE, a reduction of the RV LS was shown from the tricuspid annulus (basal segments) to the inferior free wall.

## Speckle tracking echocardiography of intracardiac echocardiography images

**Figure 4:**
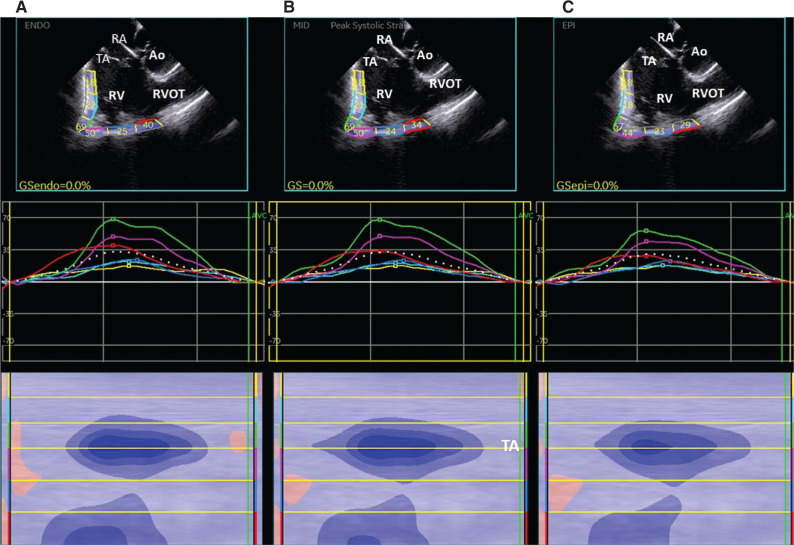
Layer-specific speckle tracking analysis applied to intracardiac images of the right ventricle. **A:** Endocardial longitudinal strain (LS). **B:** Mid-wall LS. **C:** Epicardial LS.

STE analysis on ICE views showed a reduction of the RV LS in the segments below the tricuspid valve in the 3 different myocardial layers **(Figures 4A–4C)**. Interestingly, the endocardial LS was reduced from sub-tricuspid segments to the RV apex in accordance with the fragmented potentials stored during voltage mapping. On the contrary, at the anterior RVOT wall, the unipolar voltage mapping showed fragmented potentials, and the STE analysis revealed a reduced epicardial LS.

## Catheter ablation and drug therapy

**Figure 5:**
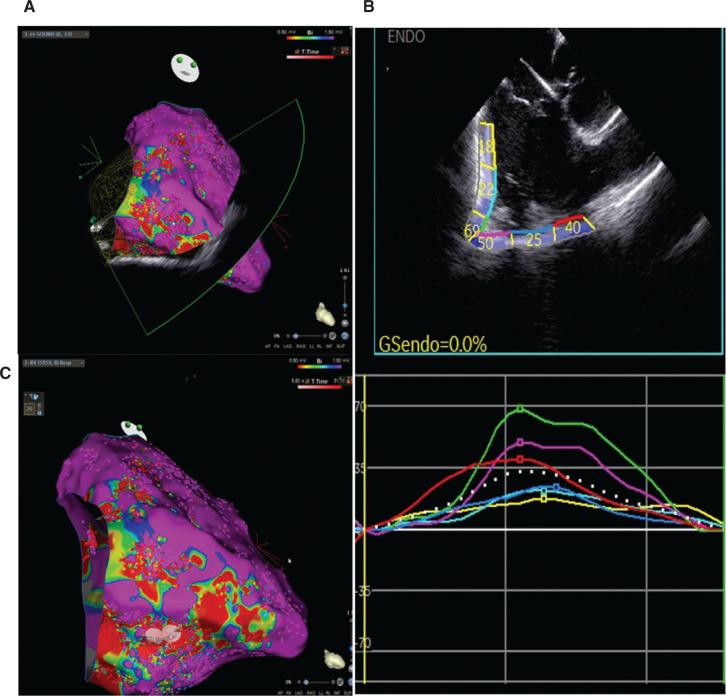
Comparison of **A:** merged ICE-voltage bipolar map with **B:** the corresponding STE analysis of ICE slice and **C:** site of RF ablation in the endocardial RV inferior wall as marked by white tags.

Considering the arrhythmic substrate in an ARVC patient, we decided to proceed with a 30-W catheter ablation using power control for a total of 2 min at the level of each fragmented potential of the inferior wall, where low voltage had been documented by unipolar and bipolar mapping **(Figure 5)**. During catheter ablation, an irritable VT occurred, and the corresponding ECG morphology was the same as that observed in the first clinical VT. Three days after catheter ablation, the patient underwent implantation of a subcutaneous implantable cardioverter-defibrillator for secondary prevention of sudden cardiac death. He was discharged 12 days after admission on a low dose of β-blocker therapy (2.5 mg of bisoprolol daily). Six months of subcutaneous implantable cardioverter-defibrillator follow-up confirmed no shock delivery.

## Discussion

ARVC still remains one of the main causes of sudden cardiac death, particularly in the younger population.^4^ In ARVC, a fibrofatty replacement involves the basal and mid-apical segments of the RV, and the so-called “triangle of dysplasia” refers to a small region involving the RV inflow tract, RVOT, and RV apex, where structural abnormalities are typically found in the early stages of the disease.^2^

TTE has been the first-line tool used in these situations, although it has low specificity. In addition to RV dimensions, indices commonly impaired are the M-mode of the tricuspid annulus in the lateral, septal, and posterior positions; tissue Doppler velocity during early and late diastole in the same positions; systolic annular velocity in the lateral annulus; and a decreased E:A ratio.^5^ Moreover, TTE indices tend to be progressively more abnormal with a likelihood of ARVC as demonstrated in adolescents.^6^

The arrhythmic substrate of ARVC can be recorded during voltage mapping as a low-amplitude, fractionated signal reflecting a pathologic conduction such that electroanatomic voltage mapping can identify the scars that correlate with pathognomonic histopathological features of the disease. The RV regional and transmural EP heterogeneities constitute the arrhythmogenic substrates, targets of catheter ablation in ARVC patients. Several studies have addressed the ability of unipolar and bipolar EAM to identify dysplastic areas identified by echocardiography in ARVC populations.

Previous studies applying speckle-tracking TTE to ARVC patients have provided promising results, with RV myocardial-deformation indices demonstrating significantly reduced strain with relevant predictive value.^7–9^ RV mechanical dispersion in ARVC can detect segments with subtle dyskinesia also in earlier stages of the disease and might reflect an arrhythmic risk.^10,11^ In particular, Kirkels et al. showed that abnormal deformation patterns and a higher mechanical dispersion, both assessed through STE parameters, are independently associated with ventricular arrhythmias in ARVC.^12^ However, compared to TTE, ICE has a better imaging resolution; moreover, it allows real-time integration with EAM and can provide an optimal RV inflow/outflow view for RV anatomy and function evaluation and strain deformation assessment.^13^

As highlighted in this clinical case, the use of ICE-derived RV strain could guide the use of EAM in order to detect and delimit with more sensitivity the areas of RV whose ablation leads to more successful results. To our knowledge, our report is the first case to describe the relationship between LS and cardiac electrograms during voltage mapping. Moreover, the use of ICE facilitates a precise definition of the RV boundaries of the tricuspid and pulmonary fibrous annuli and a reduction in fluoroscopy use for diagnostic or therapeutic EP studies and catheter ablation. However, other studies are currently needed to better characterize the combined approach of STE using ICE and voltage mapping guided by a dedicated software of reconstruction.
